# Relationships between the Regulatory Systems of Quorum Sensing and Multidrug Resistance

**DOI:** 10.3389/fmicb.2016.00958

**Published:** 2016-06-16

**Authors:** Gang-Ming Xu

**Affiliations:** Key Laboratory of Experimental Marine Biology, Institute of Oceanology, Chinese Academy of SciencesQingdao, China

**Keywords:** regulatory system, quorum sensing, multidrug resistance, transcriptional regulator, signal molecule

## Abstract

Cell–cell communications, known as quorum sensing (QS) in bacteria, involve the signal molecules as chemical languages and the corresponding receptors as transcriptional regulators. In Gram-negative bacteria, orphan LuxR receptors recognize signals more than just acylhomoserine lactones, and modulate interspecies and interkingdom communications. Whereas, in the Gram-positive *Streptomyces*, pseudo gamma-butyrolactones (GBLs) receptors bind antibiotics other than GBL signals, and coordinate antibiotics biosynthesis. By interacting with structurally diverse molecules like antibiotics, the TetR family receptors regulate multidrug resistance (MDR) by controlling eﬄux pumps. Antibiotics at subinhibitory concentration may act as signal molecules; while QS signals also have antimicrobial activity at high concentration. Moreover, the QS and MDR systems may share the same exporters to transport molecules. Among these orphan LuxR, pseudo GBL receptors, and MDR regulators, although only with low sequence homology, they have some structure similarity and function correlation. Therefore, perhaps there might be evolutionary relationship and biological relevance between the regulatory systems of QS and MDR. Since the QS systems become new targets for antimicrobial strategy, it would expand our understanding about the evolutionary history of these regulatory systems.

## Introduction

**FIGURE 1:**
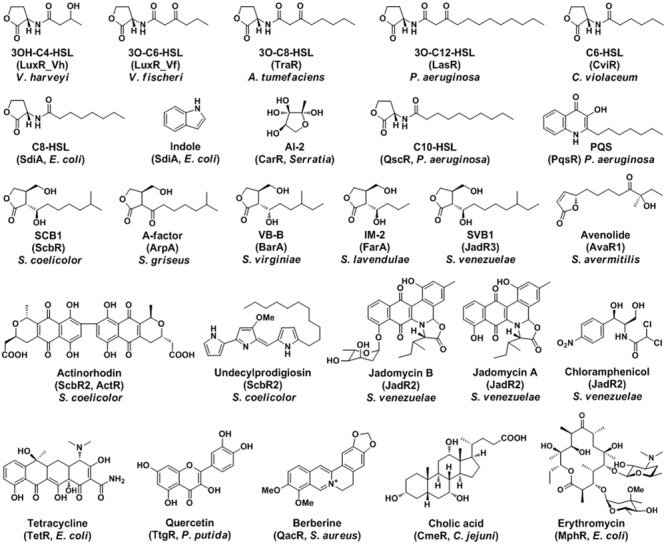
**The signal molecules and the corresponding receptors of QS and MDR in bacteria.** The representative ligands of transcriptional regulators are shown with chemical structures.

Cell–cell communications among bacteria play vital roles for their adaption and survival in the environment (Waters and Bassler, 2005). Bacteria use chemical languages (signal molecules) and corresponding receptors (transcriptional regulators) during communications (Bassler and Losick, 2006). The signal molecules are structurally diverse, including acylhomoserine lactones (AHLs), gamma-butyrolactones (GBLs), and antibiotics (Ryan and Dow, 2008). Their corresponding receptors are usually transcriptional regulators, like LuxR, GBL receptors, and TetR family (**Figure 1**). They constitute widespread signal transduction systems, such as quorum sensing (QS) system, multidrug resistance (MDR) system, and two-component regulatory system (Ramos et al., 2005). These regulatory systems all have three essential components: signal molecule, synthase, and corresponding receptor (Cuthbertson and Nodwell, 2013).

In Gram-negative bacteria, the autoinducer AHLs interact with cognate LuxR, and coordinate the bacterial quorum behaviors (Camilli and Bassler, 2006). The QS regulatory system has specific AHL synthase-receptor pairs, which possibly coevolved as functional cassettes (Gray and Garey, 2001; Lerat and Moran, 2004). However, some orphan LuxR receptors, which are not paired with synthase LuxI, have been identified later (Patankar and Gonzalez, 2009). Those LuxR homologues appeared to have different origins, which may due to horizontal gene transfer. They form various regulatory cascades and extend the bacterial regulatory networks (Patankar and Gonzalez, 2009; Subramoni and Venturi, 2009).

In Gram-positive *Streptomyces*, GBLs are used as signal molecules. By interacting with cognate receptors, GBLs activate the biosynthesis of secondary metabolites (Takano, 2006). In the GBL signaling system, cognate GBL receptors bind specific GBL molecule as ligand. The combination pairs of GBL synthase-receptor may have changed during evolution (Nishida et al., 2007). There are also some pseudo GBL receptors, whose coding genes are not adjacent to GBL synthase, and can not bind GBL molecules (Kitani et al., 2008). *Streptomyces* may reconstruct the regulatory system of secondary metabolisms for the adaption in complex habitats (Biarnes-Carrera et al., 2015).

The MDR regulatory system involves TetR family transcriptional regulators and multidrug eﬄux pumps (Paulsen, 2003). The MDR regulators can respond to structurally diverse compounds including antibiotics (Cuthbertson and Nodwell, 2013). Antibiotics abuse may lead to more eﬄux pumps, which expel various molecules (Nikaido, 2009). These molecules could act as antibiotics at high concentration, while as signals at low concentration (Romero et al., 2011). Moreover, the QS and MDR regulatory systems may share the same eﬄux pumps to transport molecules (Piddock, 2006; Martinez et al., 2009).

## Evolution of the Transcriptional Regulators of QS and MDR

The orphan LuxR homologues and their functional characterizations in Gram-negative bacteria have been summarized previously (Patankar and Gonzalez, 2009). Based on the crystal structures and regulatory roles, the LuxR homologues include two families: family-A (LuxR_Vf, TraR, SdiA, CviR, LasR, and QscR) and family-B (LuxR_Vh, HapR, and SmcR; Kim et al., 2010; Lintz et al., 2011). The two LuxR families, without virtually homology, may have different evolutionary history (Lerat and Moran, 2004).

The GBL signals and cognate receptors are commonly used in the Gram-positive *Streptomyces*. Some pseudo GBL receptors, such as BarB, FarR2, JadR2, and ScbR2, were also reported (Nishida et al., 2007). These pseudo GBL receptors not only have high sequence homology with the genuine GBL receptors (ArpA, BarA, FarA, and ScbR), but also show similarity with the MDR regulators (Cuthbertson and Nodwell, 2013). Only the crystal structure of CprB (*Streptomyces coelicolor*) was determined and found to be highly similar with these MDR regulators (TtgR, QacR, and TetR; Bhukya et al., 2014).

TetR family regulators typically act as transcriptional repressors and involve in many biological processes, such as antibiotics resistance, cell–cell communication, and regulation of metabolisms (Ramos et al., 2005). Phylogenetic analyses of the TetR family regulators suggest that these GBL receptors, pseudo GBL receptors, LuxR homologues, and MDR regulators may cluster separately (Nishida et al., 2007; Cuthbertson and Nodwell, 2013). The chemical structures of representative ligands for these transcriptional regulators are also similar within each group accordingly (**Figure 1**). The ligand-binding specificity is in good agreement with these clustered receptors. The LuxR homologues receptors only recognize AHLs in Gram-negative bacteria; while the GBL receptors only bind GBLs in Gram-positive *Streptomyces*. However, some orphan LuxR and pseudo GBL receptors recognize more than just QS signals; and the MDR regulators accept even more diverse molecules. These regulators clustered together may have high sequence homology, structure similarity, and function relevance, which might suggest some evolutionary relationships (Cuthbertson and Nodwell, 2013).

Many crystal structures of the TetR family proteins have been determined, such as TtgR, QacR, CprB, HapR, and SmcR, whose overall structures are highly similar (Schumacher et al., 2001; Alguel et al., 2007; De Silva et al., 2007; Kim et al., 2010; Bhukya et al., 2014). Structure-based multiple sequence alignment analyses suggest that these proteins have similar secondary structures, with the helix-turn-helix motif highly conserved (Ramos et al., 2005). The N-terminal DNA-binding domain has relative high conservation for specific promoters; whereas the C-terminal ligand-binding domain has more variations for diverse ligands (Nishida et al., 2007; Yu et al., 2010). The ligand-binding pocket of QS regulators is very small to ensure its binding specificity; while that of the MDR regulators is relatively large to accommodate various molecules. Structural conservation of the TetR family regulators might determine their biological relevance (Cuthbertson and Nodwell, 2013).

## Relationships Between the Regulatory Systems of QS and MDR

In Gram-negative bacteria, the LuxR receptors bind specific AHLs; whereas orphan LuxR receptors recognize signal molecules more than just AHLs, and involve in the interspecies and interkingdom communications among different bacteria and their hosts (Patankar and Gonzalez, 2009). Some orphan LuxR homologs have structural similarity with the MDR regulators of the TetR family. For example, the QS regulators HapR and SmcR, with no ligand reported, are very similar to the TetR family QacR, TtgR, and EthR (De Silva et al., 2007; Kim et al., 2010).

In Gram-positive *Streptomyces*, only several GBL molecules and cognate receptors are identified so far (Biarnes-Carrera et al., 2015). These pseudo GBL receptors can not bind endogenous GBL molecules (Nishida et al., 2007). The crystal structure of CprB was reported, with no ligand identified (Bhukya et al., 2014). Interestingly, two pseudo GBL receptors could recognize endogenous antibiotics: ScbR2 (*S. coelicolor*) respond to actinorhodin and undecylprodigiosin; JadR2 (*S. venezuelae*) binds jadomycin and chloramphenicol (Xu et al., 2010). And also, by interacting with ScbR2, the angucycline antibiotics jadomycin may function as signals to modulate the antibiotic production and morphological differentiation of *S. coelicolor* (Wang et al., 2014). The pseudo GBL receptors also negatively control the GBL biosynthesis, which expand the regulatory network (Wang et al., 2011; Liu et al., 2013). Moreover, the GBL molecule SVB1 (*S. venezuelae*) is identical to the SCB3 (*S. coelicolor*), which may suggest a novel signaling role for GBL molecules in the interspecies communication (Nodwell, 2014; Zou et al., 2014).

Microbes could produce structurally diverse natural products including antibiotics. Some investigators doubt whether the antibiotics in natural habitat can reach the killing concentration (Linares et al., 2006). Many antibiotics of subinhibitory concentration alter bacterial gene expression profiles but not inhibit bacterial growth, which is the traditional characteristics of QS signals (Davies et al., 2006; Fajardo and Martinez, 2008). Some gene expression alterations lead to significant interference in the transduction of QS signaling, which is similar to the inhibition of QS system (Linares et al., 2006). These phenomena may suggest us to rethink their physiological functions for microbes. The ecological roles of antibiotics might be as signal molecules among cell–cell communications in the natural environment (Romero et al., 2011).

The QS signals at high concentration have also been reported to have similar bioactivity like antibiotics (Schertzer et al., 2009). For instance, the 3-oxo-*N*-acylhomoserine lactones, *Pseudomonas* quinolone signal (PQS), and phenazines have been proved to have antimicrobial activities (Kaufmann et al., 2005; Dietrich et al., 2008; Schertzer et al., 2009). More and more studies have found that low concentration of antibiotics may act as signal molecules during cell–cell communication; while the QS signals also have antimicrobial activity at high concentration (Romero et al., 2011).

Moreover, the QS and MDR regulatory systems may share the same path to transport molecules. The MDR regulatory systems use the multidrug eﬄux pumps that also export QS signals (Yang et al., 2006). The drug molecules exported by these eﬄux pumps are similar to the QS signals. High concentration of antibiotics may induce overexpression of exporter genes for more eﬄux pumps. These molecules are recognized by the TetR family regulators, and these transcriptional regulators are in charge of the eﬄux transporters (Piddock, 2006; Cuthbertson and Nodwell, 2013). For example, the TtgR (*Pseudomonas putida*) regulates the eﬄux pump TtgABC, and the ActR (*S. coelicolor*) controls the exporter ActA (Alguel et al., 2007; Willems et al., 2008). The physiological role of these eﬄux pumps might be to export signal molecules for cell–cell communication (Piddock, 2006; Yang et al., 2006).

**FIGURE 2:**
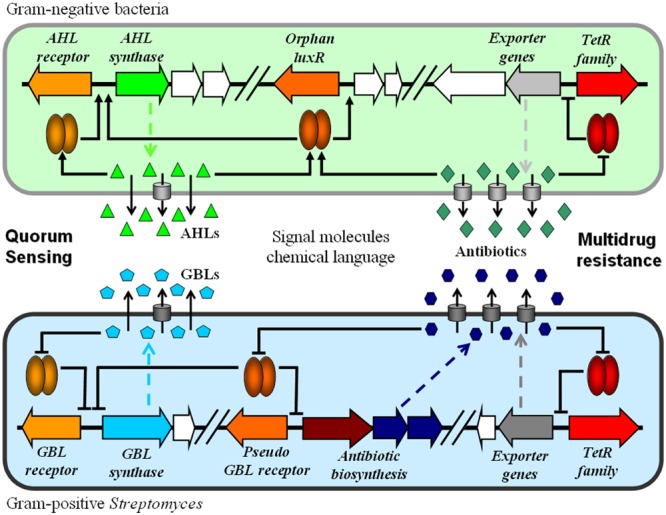
**Relationships between the regulatory systems of QS and MDR in bacteria.** Cell–cell communications involve the signal molecules (AHLs, GBLs, and antibiotics) as chemical languages and the corresponding receptors (LuxR, GBL receptor, and TetR family) as transcriptional regulators.

As summarized in **Figure 2**, the TetR family regulators play vital roles in the transcriptional regulation of cell–cell communication using chemical languages. The AHL receptors regulate the AHLs biosynthesis in Gram-negative bacteria; while the GBL receptors control the GBLs biosynthesis in Gram-positive *Streptomyces*. Some orphan LuxR and pseudo GBL receptors respond to molecules more than just QS signals. The QS signals may have antimicrobial activity; while antibiotics also act as signal molecules. The eﬄux pumps for antibiotics are also the exporters for QS signals. Therefore, the QS and MDR regulatory systems might have some evolutionary relationship and biological relevance. QS interference using inhibitors is proving to be a new strategy for antimicrobial therapy (Hirakawa and Tomita, 2013; LaSarre and Federle, 2013). Further understanding the evolutionary history and biological roles of these regulatory systems would have theoretical significance and potential application in future.

## Author Contributions

GX developed the ideas, wrote and approved the final version of the manuscript.

## Conflict of Interest Statement

The author declares that the research was conducted in the absence of any commercial or financial relationships that could be construed as a potential conflict of interest.
